# *Selaginella tamariscina* Inhibits Glutamate-Induced Autophagic Cell Death by Activating the PI3K/AKT/mTOR Signaling Pathways

**DOI:** 10.3390/ijms231911445

**Published:** 2022-09-28

**Authors:** Yun Hee Jeong, Tae In Kim, You-Chang Oh, Jin Yeul Ma

**Affiliations:** Korean Medicine (KM)-Application Center, Korea Institute of Oriental Medicine, 70, Cheomdan-ro, Dong-gu, Daegu 41062, Korea

**Keywords:** *Selaginella tamariscina*, neuroprotective effects, anti-autophagy, phosphatidylinositol 3-kinase, protein kinase B, mammalian target of rapamycin

## Abstract

Glutamate-induced neural toxicity in autophagic neuron death is partially mediated by increased oxidative stress. Therefore, reducing oxidative stress in the brain is critical for treating or preventing neurodegenerative diseases. *Selaginella tamariscina* is a traditional medicinal plant for treating gastrointestinal bleeding, hematuria, leucorrhea, inflammation, chronic hepatitis, gout, and hyperuricemia. We investigate the inhibitory effects of *Selaginella tamariscina* ethanol extract (STE) on neurotoxicity and autophagic cell death in glutamate-exposed HT22 mouse hippocampal cells. STE significantly increased cell viability and mitochondrial membrane potential and decreased the expression of reactive oxygen species, lactate dehydrogenase release, and cell apoptosis in glutamate-exposed HT22 cells. In addition, while glutamate induced the excessive activation of mitophagy, STE attenuated glutamate-induced light chain (LC) 3 II and Beclin-1 expression and increased p62 expression. Furthermore, STE strongly enhanced the phosphatidylinositol 3-kinase (PI3K)/protein kinase B (Akt)/mammalian target of rapamycin (mTOR) phosphorylation activation. STE strongly inhibited glutamate-induced autophagy by activating the PI3K/Akt/mTOR signaling pathway. In contrast, the addition of LY294002, a PI3K/Akt inhibitor, remarkably suppressed cell viability and *p*-Akt and p62 expression, while markedly increasing the expression of LC3 II and Beclin-1. Our findings indicate that autophagy inhibition by activating PI3K/Akt/mTOR phosphorylation levels could be responsible for the neuroprotective effects of STE on glutamate neuronal damage.

## 1. Introduction

Oxidative stress and excitotoxicity dysfunction are important causes of several neurodegenerative diseases, including Alzheimer’s disease, Parkinson’s disease, and brain ischemia [1,2,3,4]. Glutamate, an excitatory neurotransmitter in the central nervous system, is closely linked to cell survival, migration, and differentiation during brain development. On the contrary, an excessive amount of glutamate can overstimulate its receptors and induce calcium overload, resulting in neuronal cell damage and death via oxidative stress or excitotoxicity [5,6]. Brain tissue is highly vulnerable to reactive oxygen species (ROS) during oxidative stress. In addition, ROS are closely related to mitochondrial dysfunction with the loss of the mitochondrial membrane potential (MMP) [7]. The overproduction and accumulation of ROS by mitochondrial damage or dysfunction of the antioxidant system can contribute to cell destruction and death [8,9,10]. Therefore, a reduction of ROS accumulation might be effective in treating neurodegenerative diseases.

Autophagy is an intracellular catabolism process involved in the clearing and recycling of damaged, unnecessary, or dysfunctional cell components through a lysosome-dependent regulated mechanism. It plays a crucial role in normal neuron function by managing cell protein degradation and damaged organelles fused with lysosomes, providing energy, and promoting cell survival [11,12]. Previous studies have demonstrated that autophagy protects brain tissue against damage caused by neurodegenerative diseases [13,14,15,16,17,18]. However, overstimulated autophagy can also trigger cell dysfunction or autophagy-like cell death by damaging normal organelles [19]. When autophagy is activated, a cytosolic form of light chain 3 (LC3), (LC3 I), is conjugated to phosphatidyl ethanolamine to form LC3 II, which is recruited to autophagosomal membranes. Therefore, the LC3 II/LC3 I ratio is usually adopted to reflect the number of autophagosomes and the level of autophagy in cells [20]. In addition, the mammalian target of rapamycin (mTOR) is an important autophagy modulator that receives inputs from different signaling pathways. mTOR is primarily regulated by the phosphatidylinositol 3-kinase (PI3K)/protein kinase B (Akt) signaling pathways, which are activated by neurotrophin receptors and growth factors that promote cell proliferation and cell survival [21,22]. Furthermore, Beclin-1 is a specific autophagy gene that interacts with the B-cell lymphoma 2 (Bcl-2) family of anti-apoptotic proteins and exhibits an inhibitory effect on autophagy [23]. Therefore, the inhibition of autophagy to an appropriate level and the modulation of related mechanisms may be suggested as potential targets for treating or preventing neurodegenerative diseases.

*Selaginella tamariscina* (ST) is a perennial evergreen plant known as Kwon Baek or Boo Cheo Son. It is mainly distributed in East Asia and is a well-known herb in traditional oriental medicine, used to treat gastrointestinal bleeding, hematuria, leucorrhea, inflammation, chronic hepatitis, gout, and hyperuricemia [24,25,26]. Recent studies have shown that ST has biological activities and anti-inflammatory, anti-cancer, anti-fungal, anti-diabetic, anti-bacterial, and anti-allergic pharmacological functions [27,28,29]. However, the neuroprotective effects of *Selaginella tamariscina* ethanol extract (STE) on the glutamate-exposed mouse hippocampal neuron cell line remain unknown. We screened over 300 hot water and ethanol extracts stored in our herbal extract bank to find effective substances that effectively prevent neurotoxicity, and STE, which showed excellent efficacy, was selected as the test substance. In addition, the effects of STE on the expression of several representative mechanistic proteins were preliminarily evaluated, and based on the results, it was possible to hypothesize that STE inhibits neurotoxicity by regulating oxidative stress and autophagy. Therefore, in this study, the pharmacological action of STE on glutamate-induced oxidative stress and neurotoxicity is investigated using mouse HT22 hippocampal cells. Moreover, its influence on potential mechanisms, including the regulation of autophagy, is examined.

## 2. Results

### 2.1. STE Attenuates Glutamate-Induced Cytotoxicity in HT22 Cells

**Figure 1:**
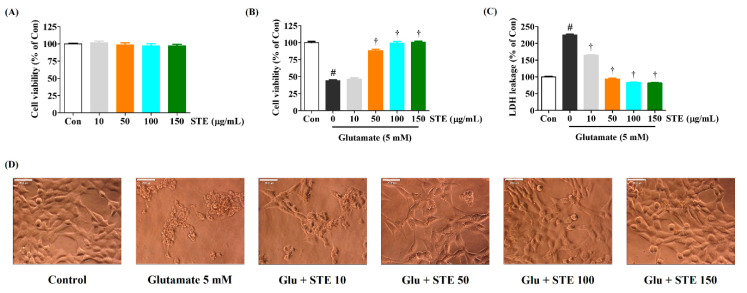
Effects of *Selaginella tamariscina* ethanol extract (STE) on glutamate-induced cytotoxicity in HT22 cells. (**A**) HT22 cells were incubated with STE concentrations of 10, 50, 100, or 150 μg/mL. (**B**–**D**) After STE pretreatment concentrations of 10, 50, 100, or 150 μg/mL, HT22 cells were stimulated with glutamate (5 mM). (**D**) Images represent the three independent experiments at a magnification of 400×. Scale bar = 20 μm. Control cells were incubated with the vehicle alone. Data are presented as mean ± standard error of the mean of three independent experiments. Con, control; LDH, lactate dehydrogenase; Glu, glutamate. Statistical significance was defined as # *p* < 0.05 (vs. control) and † *p* < 0.001 (vs. glutamate).

CCK assay results show that STE (10–150 μg/mL) did not have any toxic effect in HT22 cells (Figure 1A). Thus, this study used STE at 50–150 μg/mL. Our data show that, compared with the non-treated control, cell viability was reduced by approximately 52% in the glutamate-alone-treated cells. Meanwhile, a pretreatment of STE concentration-dependently enhanced cell viability. At an STE concentration of 150 μg/mL, cell viability increased to approximately 96%, a level similar to the control cells (Figure 1B). Further study of changes in the intracellular release of LDH showed that the glutamate treatment increased LDH leakage by approximately 225% compared with non-treated control cells. Leakage was significantly reduced in a concentration-dependent manner by pretreatment with different STE concentrations (50, 100, or 150 μg/mL) (Figure 1C). In addition, because cell morphology was examined with an optical microscope, glutamate caused nuclear condensation and cell shrinkage. In contrast, STE pretreatment showed a concentration-dependent effect in maintaining cell structure and inhibiting morphological changes (Figure 1D). Therefore, STE significantly alleviates neurotoxicity in glutamate-exposed HT22 cells.

### 2.2. STE Reduced Intracellular ROS Produced by Glutamate

**Figure 2:**
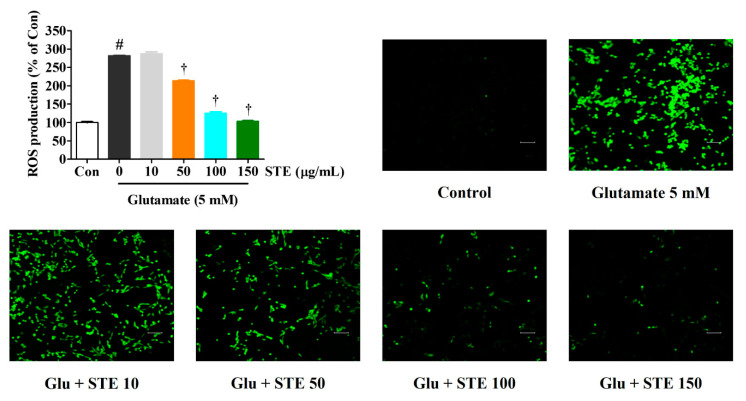
Effects of *Selaginella tamariscina* ethanol extract (STE) against glutamate-induced intracellular reactive oxygen species (ROS) production in HT22 cells. Cells were pretreated with STE concentrations of 10, 50, 100, or 150 μg/mL, and then with 5 mM glutamate. H_2_DCFDA (20 μM), an oxidation-sensitive fluorescence dye, was used to assess the ROS levels. The expression of ROS was determined using a fluorescence microscope and fluorescence microplate reader. Scale bar = 200 μm. Control cells were incubated with the vehicle alone. All experiments were repeated at least three times, and similar results were obtained. Data are presented as mean ± standard error of the mean. Con, control; Glu, glutamate. Statistical significance was defined as # *p* < 0.05 (vs. control) and † *p* < 0.001 (vs. glutamate).

Excessive ROS production can cause oxidative stress and mitochondrial dysfunction, resulting in cell apoptosis. Thus, we determined ROS production in HT22 cells using H_2_DCFDA, a fluorescent ROS indicator. As shown in Figure 2, glutamate alone significantly increased intracellular ROS production, whereas pretreatment with STE effectively diminished ROS generation in a concentration-dependent manner. The fluorescent images in Figure 2 also show an effect similar to that mentioned above, indicating that STE protects dopaminergic neurons from glutamate-induced oxidative stress.

### 2.3. STE Inhibited Glutamate-Induced Neuronal Cell Death

**Figure 3:**
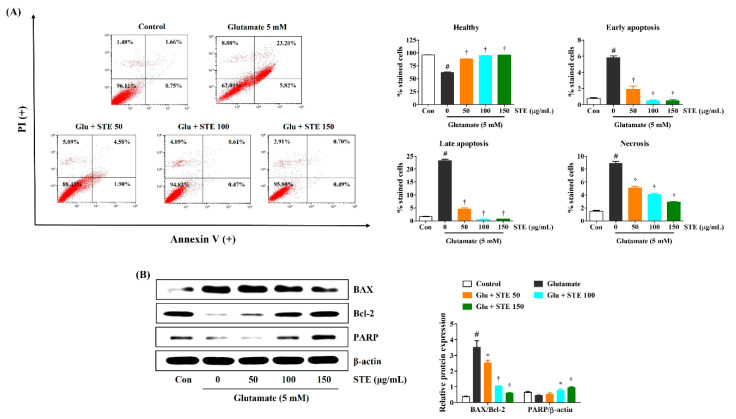
Effects of *Selaginella tamariscina* ethanol extract (STE) against glutamate-induced apoptosis in HT22 cells. Cells were pretreated with STE concentrations of 50, 100, or 150 μg/mL and then exposed to glutamate (5 mM). (**A**) Apoptosis of HT22 cells was evaluated via flow cytometry. Quantitative data showed the percentage of healthy, early apoptotic, late apoptotic, and necrotic cells according to treatment. (**B**) The expression levels of Bcl-2-associated X, B-cell lymphoma 2, and Poly (ADP-ribose) polymerase were determined via Western blot analysis. Control cells were incubated with the vehicle alone. Blot images represent three independent experiments. Data are presented as mean ± standard error of the mean. PI, propidium iodide; Glu, glutamate; Con, control; BAX, Bcl-2-associated X; Bcl-2, B-cell lymphoma 2; PARP, Poly (ADP-ribose) polymerase. Statistical significance was defined as # *p* < 0.05 (vs. control), * *p* < 0.05, and † *p* < 0.001 (vs. glutamate).

The protection from glutamate-induced apoptotic neurotoxicity conferred by STE pretreatment was measured by flow cytometry. As shown in the flow cytometry results using Annexin V, glutamate significantly increased apoptotic bodies in HT22 cells. In contrast, STE pretreatment resulted in a marked decrease in several apoptotic and necrotic bodies. Moreover, when the cells were treated with 100 or 150 μg/mL, STE protected the cells similar to the vehicle of the control (Figure 3A). Additionally, we measured the protein expression related to neuronal cell death, including Bcl-2, Bcl-2-associated X (BAX) and Poly (ADP-ribose) polymerase (PARP), via Western blotting. As shown in Figure 3B, compared with control cells, exposure to glutamate resulted in the upregulation of BAX and the downregulation of Bcl-2 in HT22 hippocampal cells. Meanwhile, STE pretreatment markedly decreased the expression of BAX and increased the expression of Bcl-2 and PARP in a concentration-dependent manner (Figure 3B). These results suggest that STE pretreatment exerts neuroprotective effects by inhibiting glutamate-induced apoptotic and necrotic cell death.

### 2.4. STE Prevents Glutamate-Induced Loss of MMP

**Figure 4:**
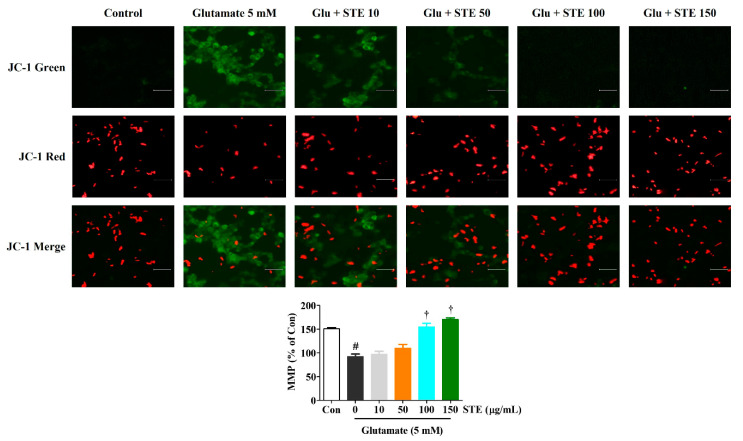
Effects of *Selaginella tamariscina* ethanol extract (STE) on glutamate-induced mitochondrial dysfunction in HT22 cells. The mitochondrial membrane potential (MMP) was assessed by microscopy using JC-1 staining. Images represent three independent experiments at a magnification of 200×. Scale bar = 100 μm. Red fluorescence indicated normal MMP; green fluorescence indicated damaged mitochondria with MMP loss. The histogram shows the red/green fluorescence intensity ratio. Control cells were incubated with the vehicle alone. Data are presented as mean ± standard error of the mean of three independent experiments. Glu, glutamate; Con, control. Statistical significance was defined as # *p* < 0.05 (vs. control) and † *p* < 0.001 (vs. glutamate).

MMP was determined with a mitochondrial-specific cationic dye, JC-1, to explore the effects of STE on mitochondrial functions. In normal cells, JC-1 aggregated with healthy mitochondria and exhibited red fluorescence. JC-1 existed in monomer form in the cytoplasm, and exhibited green fluorescence in the cells with depolarized mitochondria. As shown in Figure 4, the control cells exerted high red and low green fluorescence. However, the green fluorescence intensity markedly increased after glutamate treatment, while red fluorescence intensity decreased. The results show that STE pretreatment could effectively prevent the MMP of HT22 cells in a concentration-dependent manner, as shown by the decreased green and increased red fluorescence (Figure 4). These results reveal that STE prevents MMP loss caused by glutamate-induced oxidative stress in HT22 cells.

### 2.5. STE Inhibited Glutamate-Induced Autophagy and Downregulated the Related Molecular Mechanism in HT22 Cells

**Figure 5:**
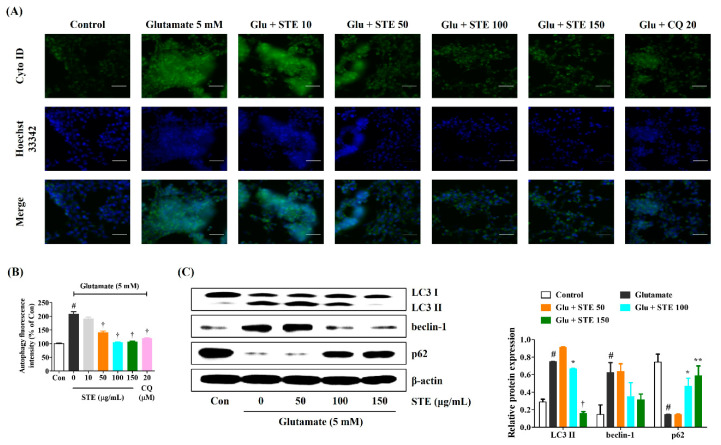
Effects of *Selaginella tamariscina* ethanol extract (STE) against glutamate-induced autophagy in HT22 cells. Cells were pretreated with STE concentrations of 10, 50, 100, or 150 μg/mL or 20 μM chloroquine and then exposed to glutamate (5 mM). (**A**) The autophagy level of HT22 cells was evaluated using Cyto-ID^®^ green autophagy dye and analyzed by fluorescence microscopy. Images represent three independent experiments at a magnification of 200×. Scale bar = 100 μm. (**B**) Autophagy fluorescence intensity level was determined using Image J software. (**C**) The expression levels of light chain 3 II, Beclin-1, and p62 were determined via Western blot analysis. Control cells were incubated with the vehicle alone. Blot images were representative of the three independent experiments. Data are presented as mean ± standard error of the mean. Glu, glutamate; CQ, chloroquine; Con, control; LC, light chain. Statistical significance was defined as # *p* < 0.05 (vs. control), * *p* < 0.05, ** *p* < 0.01, and † *p* < 0.001 (vs. glutamate).

In HT22 cells, autophagy induction by glutamate and regulatory efficacy by STE pretreatment were measured using fluorescence microscopy and a Cyto-ID autophagy detection kit. As can be seen from the change in the Cyto-ID positive cells (GFP) level, glutamate sharply increased autophagy in HT22 cells, and pretreatment with STE reduced autophagy in a concentration-dependent manner (Figure 5A,B). In addition, the autophagy inhibitor chloroquine was used as a positive control, and relatively excellently inhibited autophagy of HT22 cells (Figure 5A,B). Next, we investigated the expression of related proteins by Western blotting to elucidate the molecular mechanisms involved in autophagy inhibition of STE in glutamate-exposed HT22 cells. Glutamate treatment resulted in a significant increase in the expression of the autophagy marker proteins LC3 II and Beclin-1 compared with the control cells, but notably resulted in the decreased expression of the autophagic/lysosomal degradation protein p62, as shown in Figure 5C. In contrast, STE pretreatment effectively reduced the expression of LC3 II and Beclin-1 while notably increasing the level of p62, as shown in Figure 5C. These results suggest that STE exerts neuroprotective effects by blocking the activation of glutamate-induced excessive autophagy and regulating the relevant molecular mechanisms in HT22 cells.

### 2.6. STE Prevented Glutamate-Induced Autophagy via the Activation of the PI3K/Akt/mTOR Signaling Pathway

**Figure 6:**
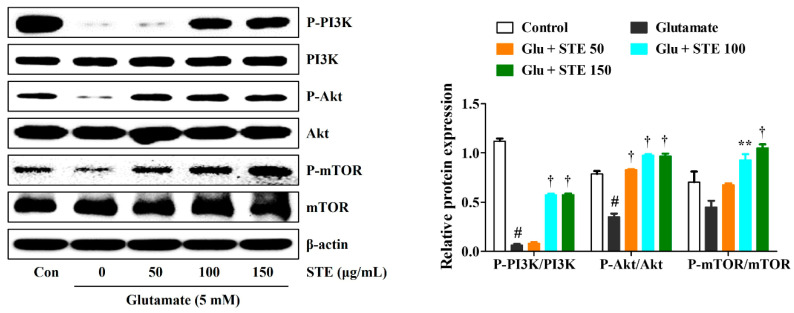
Effects of *Selaginella tamariscina* ethanol extract (STE) on the phosphorylation of phosphoinositide 3-kinase, protein kinase B, and mammalian target of rapamycin in glutamate-exposed HT22 cells. Cells were pretreated with STE concentrations of 50, 100, or 150 μg/mL and then exposed to glutamate (5 mM). Control cells were incubated with the vehicle alone. Blot images represent the three independent experiments. Data are expressed as mean ± standard error of the mean. Con, control; PI3K, phosphoinositide 3-kinase; Akt, protein kinase B; mTOR, mammalian target of rapamycin; Glu, glutamate. Statistical significance was defined as # *p* < 0.05 (vs. control), ** *p* < 0.01, and † *p* < 0.001 (vs. glutamate).

Previous studies have shown that PI3K/Akt/mTOR signaling is one of the key pathways closely associated with cell survival and the regulation of autophagy [20,21]. Thus, the current study investigated whether STE could affect glutamate-induced autophagy through the PI3K/Akt/mTOR signaling pathway. As shown in Figure 6, the expression levels of P-PI3K, P-Akt, and P-mTOR were strikingly reduced in the glutamate-exposed hippocampal cells compared with the control. However, STE pretreatment significantly upregulated the phosphorylation of PI3K/Akt/mTOR compared with the glutamate alone-treated HT22 cells (Figure 6). Hence, STE protected HT22 cells from glutamate-induced neurotoxicity by inhibiting autophagy.

### 2.7. LY294002 Inhibits the Neuroprotective Effects of STE

**Figure 7:**
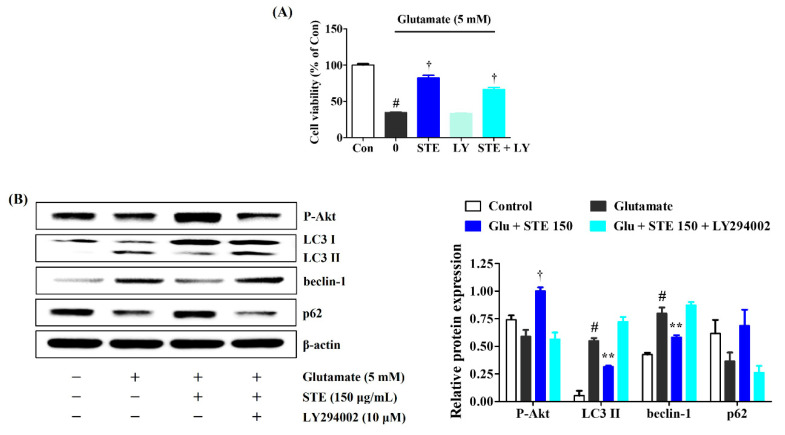
Suppressive effects of LY294002 on the neuroprotective action of *Selaginella tamariscina* ethanol extract (STE). HT22 cells were incubated with or without STE combined with LY294002 before glutamate treatment. (**A**) Cell viability and (**B**) the expression of phospho-protein kinase B, light chain 3 II, Beclin-1, and p62 were assessed using a cell-counting kit and Western blot, respectively. Control cells were incubated with the vehicle alone. Blot images represent three independent experiments. Data are presented as mean ± standard error of the mean. Con, control; LY, LY294002; Akt, protein kinase B; LC, light chain; Glu, glutamate. Statistical significance was defined as # *p* < 0.05 (vs. control), ** *p* < 0.01, and † *p* < 0.001 (vs. glutamate).

We used the PI3K/Akt inhibitor LY294002 to investigate whether STE affects glutamate-induced autophagy through PI3K/Akt pathway regulation. As shown in Figure 7, additional treatment with LY294002 markedly decreased the neuroprotective effects of STE on glutamate-induced cell death. In addition, the expression levels of P-Akt and p62 were significantly reduced. In contrast, the expression of LC3 II and Beclin-1 was markedly increased. These results again prove that STE affects PI3K/Akt phosphorylation levels, thereby reducing glutamate-induced autophagy.

### 2.8. Identification and Quantitative Analysis of the Chemical Constituents of STE

**Figure 8:**
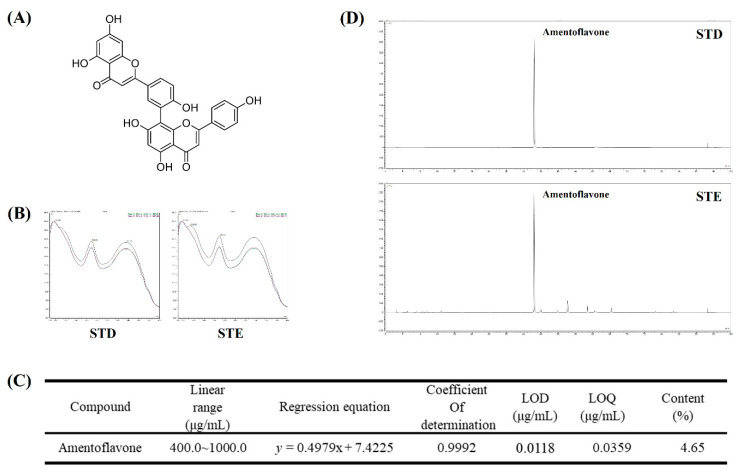
HPLC chromatogram of *Selaginella tamariscina* ethanol extract (STE). (**A**) Chemical structure of amentoflavone. (**B**) UV spectra of standard compound of amentoflavone and STE. (**C**) Regression equations, limit of detection, and limit of quantitation of amentoflavone. (**D**) HPLC chromatogram of amentoflavone standard solution and STE. STD, standard; LOD, limit of detection; LOQ, limit of quantitation.

We conducted HPLC analysis to identify and quantify the compound that showed the bioactivity of STE. The compound in STE was identified as amentoflavone, a standard component used for ST verification tests according to the Korean pharmacopeia. STE HPLC chromatogram and amentoflavone were detected in our HPLC conditions (tR 43.00 min, Figure 8). The content of amentoflavone was confirmed. The mean value of amentoflavone in the STE HPLC chromatogram was calculated using a standard curve prepared from a diluted stock solution of amentoflavone at 1000 ppm. The value was 4.65% (Figure 8).

## 3. Discussion

Oxidative stress due to ROS is one of the key causes of neurodegenerative diseases, including Alzheimer’s disease, Parkinson’s disease, and cerebral ischemia [1,2,3,4]. Several studies have indicated that prolonged oxidative stress is closely related to autophagy processes, leading to autophagic cell death in the neuronal system [4,30]. Although autophagy is generally neuroprotective, recent reports have demonstrated that excessive levels of autophagy may exacerbate cell damage and result in autophagic cell death or apoptosis [19,31]. It has also been reported that the inhibition of autophagy can restore neurodegenerative damage [32]. Therefore, reducing glutamate-induced oxidative damage and neuronal cell death via regulation of autophagy processes may be useful for preventing and treating neurodegenerative diseases.

ST is a medicinal herb with various physiological effects, including anti-inflammatory, anti-cancer, anti-fungal, anti-diabetic, anti-bacterial, and anti-allergic activity [27,28,29]. Nevertheless, the protective effects of STE against glutamate-mediated neurotoxicity in neuronal cells have not yet been studied. Therefore, the current study investigated the therapeutic potential and underlying mechanism of STE against glutamate-induced oxidative stress and neuronal cell death by regulating autophagy in a mouse hippocampal neuron cell line.

This study showed that the viability and proliferation of HT22 cells were significantly reduced while LDH release and cell apoptosis were elevated with 5 mM of glutamate. However, STE pretreatment improved cell viability and suppressed LDH release and cell apoptosis in glutamate-exposed HT22 cells (Figure 1). In addition, STE markedly attenuated the overproduction of ROS caused by glutamate treatment in a concentration-dependent manner (Figure 2). The accumulation of ROS by glutamate stimulation was linked to the mitochondrial membrane lipids response, contributing to mitochondrial permeability changes and dysfunction caused by MMP reduction, resulting in cell destruction and death [8,9]. Our results indicate that STE pretreatment effectively blocks the MMP reduction caused by glutamate-induced oxidative stress (Figure 4). Moreover, STE notably inhibited the mitochondrial apoptotic factors, including BAX and cleaved-PARP, and significantly increased the expression of anti-apoptotic factors, including Bcl-2 and PARP (Figure 3). Thus, STE protected neuronal cells by inhibiting the overproduction of ROS and restoring mitochondrial function exposed to glutamate.

LC3 is an important marker of the occurrence of autophagy. It exists in two forms: LC3 I and LC3 II. During autophagy, the cytosolic form LC3 I is further conjugated to an autophagosome-associated form (LC3 II), which is recruited to autophagosomal membranes. Thus, an increase in LC3 II levels has been widely used to indicate the activation of autophagy [33]. Beclin-1 is a positive regulator of autophagy, and examines the degree of autophagy index. It might mediate other autophagic proteins attached to autophagosomal membranes and reduce LC3 II accumulation. P62, which interacts with LC3, enters the autophagosome and is degraded by lysosomes. The changes in the expression of LC3, Beclin-1, and p62 protein are widely used to indicate autophagy flux. Our experimental results revealed that the expression of LC3 II and Beclin-1 increased, whereas the level of p62 was suppressed under glutamate-treated HT22 cells. Interestingly, STE pretreatment strongly reduced the expression of LC3 II and Beclin-1 while dramatically enhancing the expression of p62 (Figure 5). These data demonstrated that STE might effectively suppress excessive autophagy induced by glutamate in HT22 cells.

PI3K/Akt/mTOR signaling is an important pathway closely linked to cell survival and the regulation of autophagy [20,21]. Hence, we further determined the underlying mechanism of the neuroprotective effects of STE on glutamate-induced autophagy in HT22 cells. The current study measured the effects of STE on the activation of the PI3K/Akt/mTOR pathway. The activation of PI3K/Akt downstream can phosphorylate mTOR, a pivotal autophagy regulator [34]. Western blotting results implied that STE pretreatment concentration-dependently upregulated the phosphorylation of PI3K/Akt/mTOR compared with the glutamate-alone-treated HT22 cells (Figure 6).

In contrast, the inclusion of LY294002, a PI3K/Akt, inhibited the neuroprotective effects of STE in glutamate-exposed HT22 cells. Moreover, LY294002 combined with STE markedly blocked P-Akt and p62 expression, while LC3 II and Beclin-1 expression significantly increased with glutamate stimulation (Figure 7). Therefore, STE may have neuroprotective effects by inhibiting glutamate-induced excessive autophagy by activating the PI3K/Akt signaling pathways.

We performed phytochemical analyses using HPLC to investigate the relationships between the physiological activities of STE and its components. As a result, amentoflavone was identified as the main component of STE. Previous studies have shown that amentoflavone ameliorates memory deficits and abnormal autophagy by regulating mTOR signaling [35]. Moreover, it exerts neuroprotective effects by inhibiting NLRP3 inflammasome [36]. In addition, amentoflavone protects hippocampal neurons through antioxidative and anti-apoptotic effects [37]. Based on the current HPLC analysis and previous studies about amentoflavone, the anti-autophagy properties and neuroprotective effects of STE can likely reflect the presence of amentoflavone.

## 4. Materials and Methods

### 4.1. Materials and Reagents

Dulbecco’s modified Eagle’s medium (DMEM), antibiotics, and fetal bovine serum (FBS) were obtained from Hyclone Laboratories (Logan, UT, USA). All cell culture dishes and plates were obtained from SPL Life Sciences (Pocheon, Korea). Bovine serum albumin (BSA) and dimethyl sulfoxide were obtained from Sigma-Aldrich (St. Louis, MO, USA). The cell-counting kit (CCK) and the lactate dehydrogenase (LDH) assay were purchased from Dojindo Molecular Technologies (Kumamoto, Japan) and Abcam, Inc. (Cambridge, UK), respectively. 2,7-dichlorohydrofluorescein diacetate (H_2_DCFDA) was obtained from Invitrogen (Carlsbad, CA, USA). The Annexin V-FITC/propidium iodide (PI) apoptosis detection kit was obtained from BD Biosciences (Franklin Lakes, NJ, USA). The CYTO-ID^®^ autophagy detection kit (ENZ-51031-K200) was purchased from Enzo Life Sciences (Farmingdale, NY, USA). The polyvinylidene difluoride (PVDF) membrane was acquired from Millipore (Bedford, MA, USA). Primary antibodies and horseradish peroxidase (HRP)-conjugated secondary antibodies were purchased from Cell Signaling Technology, Inc. (Boston, MA, USA) and Santa Cruz Biotechnology (Santa Cruz, CA, USA). The standard constituent, amentoflavone, used in high-performance liquid chromatography (HPLC) analysis was purchased from Sigma–Aldrich. HPLC grade acetonitrile was obtained from Merck KFaA (Darmstadt, Germany) and formic acid from Sigma–Aldrich. Triple filtered water was prepared with the Puris-Evo-UP water system with Evo-UP Dio VFT and Evo-POP Dico 20 (Mirae ST Co., Ltd., Anyang, Korea).

### 4.2. STE Preparation

Dried ST was purchased from YeongcheonHyundai Herbal Market (Yeongcheon, Korea) and authenticated by Professor Ki Hwan Bae of the College of Pharmacy, Chungnam National University (Daejeon, Korea). Aerial parts of ST (50 g) were extracted in 70% ethanol (390 mL) at 40 °C in a shaking incubator (100 rpm) for 24 h. The extract solution was then filtered and concentrated using a rotary vacuum evaporator (Buchi, Tokyo, Japan). Samples were freeze-dried and stored in a desiccator at −20 °C before use.

### 4.3. Cell Culture

The HT22 mouse hippocampal neuronal cell line was cultured in a DMEM medium containing 10% FBS and 1% antibiotics at 37 °C under a 95% air/5% CO_2_ atmosphere. The culture medium was renewed every 2 days, and cells were grown to 80–85% confluence for the experiments.

### 4.4. Measurement of Cell Viability

HT22 cell survival was assessed using a CCK assay following the manufacturer’s protocol in a 96-well culture plate (5.0 × 10^3^ cells/100 μL/well). The cells were treated with 5 mM glutamate in the presence or absence of STE (10, 50, 100, or 150 μg/mL). After 24 h, 10 μL of CCK reagent was added to each well and incubated for 1 h. The absorbance of each well at 450 nm was measured using a microplate reader (SpectraMax i3, Molecular Devices, San Jose, CA, USA).

### 4.5. Cytotoxicity Assay

Cytotoxicity was analyzed by measuring the LDH enzyme level released by dead cells in the culture medium; 5.0 × 10^3^ cells were cultured in a 96-well plate at a volume of 100 μL per well. After treatment with 5 mM glutamate in the presence or absence of STE (50, 100, or 150 μg/mL) for 24 h, the supernatant was obtained by centrifugation, and 10 μL of the supernatant was transferred to a new 96-well plate. The reaction solution (100 μL/well) was added to each well, and LDH activity was calculated from the decrease in absorbance at 450 nm. Data represent the percentage of LDH release relative to non-treated control cells.

### 4.6. Measurement of Intracellular ROS Levels

The intracellular ROS level was measured with H_2_DCFDA. After STE and glutamate treatment, 10 μM H_2_DCFDA was added, and cells were incubated at 37 °C for 30 min in darkness. The stained cells were washed twice with ice-cold phosphate-buffered saline (PBS). Fluorescence was estimated using a microplate reader at an excitation wavelength of 485 nm and an emission wavelength of 525 nm. Fluorescent images were obtained using a fluorescence microscope (Eclipse Ti, Nikon, Tokyo, Japan).

### 4.7. Measurement of the MMP

The MMP was measured by staining with JC-1. After STE and glutamate treatment, the cells were stained with a JC-1 working solution (5 μg/mL) for 15 min at 37 °C in darkness. After washing with PBS, the cells were mounted and analyzed by spectrofluorometry (SpectraMax i3) at 490 nm for excitation and 530 nm for the emission of green (monomer form) fluorescence and 520 nm for excitation and 590 nm for the emission of red (aggregate form) fluorescence. The representative fluorescence images were obtained using a fluorescence microscope.

### 4.8. Cell Death Assessment by Flow Cytometry

A FITC-Annexin V apoptosis detection kit was used according to the manufacturer’s instructions to quantify apoptosis. The cells were harvested 24 h after exposure to glutamate and stained with PI and FITC conjugated Annexin V for 15 min at room temperature in darkness. The stained cells were analyzed using flow cytometry (FACSCalibur, BD Biosciences).

### 4.9. Cyto-ID Autophagy Detection Assay

The autophagic flux was monitored by a Cyto-ID^®^ autophagy detection kit according to the manufacturer’s recommendations. In brief, the cells were washed with PBS supplemented with 5% FBS and CYTO-ID^®^ green detection reagent and Hoechst 33342 nuclear stain were added into PBS supplemented with 5% FBS. The cells were then incubated at 37 °C for 30 min in the dark and then washed twice with PBS. Fluorescent images were obtained using a fluorescence microscope (Eclipse Ti, Nikon, Tokyo, Japan).

### 4.10. Nuclear and Cytosolic Protein Extraction

Cytosolic and nuclear proteins were fractionated using NE-PER Nuclear and Cytoplasmic Extraction Reagents (Thermo Fisher, Rockford, IL, USA) following the manufacturer’s instructions.

### 4.11. Western Blot Analysis

Cells were collected after STE and glutamate treatment, rinsed, and lysed in radioimmunoprecipitation assay lysis buffer (Millipore) containing protease and phosphatase-inhibitor cocktails (Roche, Basel, Switzerland). The lysate was centrifuged for 10 min at 14,000 *g*, and the supernatant was collected. The protein concentration was determined using Bradford’s reagent. Total proteins were separated using sodium dodecyl sulfate-polyacrylamide gel electrophoresis and electrically transferred to a PVDF membrane. The membranes were blocked in 3% BSA and incubated with their respective primary antibodies overnight at 4 °C. The membranes were washed thrice with tris-buffered saline with 0.1% Tween 20 (TBS-T) for 10 min each time. Then, the membranes reacted with HRP-labeled secondary antibodies for one h at room temperature, followed by cleaning thrice with TBS-T for 10 min each time. The relative intensity of protein expression was quantitated using a ChemiDoc^TM^ Touch Imaging System (Bio-Rad, Hercules, CA, USA). The relative protein expression was determined using Image J software (version 1.53k) with normalization to the control value. Table 1 shows information regarding various primary and secondary antibodies.

### 4.12. Preparation of STE and Standard Solution

STE was accuracy-weighed and dissolved in methanol at a 10 mg/mL concentration. The standard stock solution was prepared at 1 mg/mL (1000 ppm) and then diluted with methanol at each concentration to make a standard curve. All prepared solutions were kept at 4 °C before use. The STE sample and standard stock solution were filtered through a 0.2 μm PTFE membrane syringe filter, and then 10 μL of the filtrate was injected for HPLC analysis.

### 4.13. HPLC Conditions

The HPLC was a Dionex ultimate 3000 system consisting of a column oven, an autosampler, a binary pump, a diode array UV/Vis detector used to analyze STE, and the amentoflavone constituent. The detailed conditions were eluted solvent 0.1% formic acid (A) and acetonitrile (B), flow rate 1.0 mL/min, the gradient method, 0–10 min 3–15% B; 10–50 min 15–50% B; 50–80 min 50–100% B, was applied. A Luna C18 column (250 mm × 4.6 mm, 5 μm, Phenomenex) was used with a column temperature of 40 °C and an injection volume of 10 μL. The UV-absorption detection level was 340 nm. All HPLC chromatograms were processed using Chromeleon software.

### 4.14. Statistical Analysis

The data are presented as the mean ± standard error of the mean for three independent experiments. The statistical analysis was performed using GraphPad Prism version 5.02 (GraphPad Software, Inc., San Diego, CA, USA). Data were analyzed by one-way analysis of variance, followed by the Dunnett’s test, after comparing each sample. A *p*-value < 0.05 represented a statistical significance.

## 5. Conclusions

In conclusion, this study revealed that STE alleviates glutamate-induced cytotoxicity in HT22 cells via the inhibition of ROS accumulation, apoptosis, and mitochondrial dysfunction. Furthermore, STE exhibits neuroprotective effects in glutamate-exposed hippocampal cells by inhibiting autophagy through the activation of the PI3K/Akt/mTOR signaling pathway. Moreover, amentoflavone, a main component of STE, may closely correlate with the anti-autophagy properties and neuroprotective effects of STE. Therefore, STE may be a potential candidate for preventing and treating neurodegenerative diseases.

## Figures and Tables

**Table 1:** Primary and secondary antibodies use for Western blot analysis.

Antibody	Corporation	Product No.	RRID	Dilution Rate
BAX	Cell Signaling	#2772	AB_10695870	1:1000
Bcl-2	Cell Signaling	#3498	AB_1903907	1:1000
PARP	Cell Signaling	#9532	AB_659884	1:1000
β-actin	Cell Signaling	#4970	AB_2223172	1:1000
LC3	Cell Signaling	#4108	AB_2137703	1:1000
beclin-1	Cell Signaling	#3738	AB_490837	1:1000
p62	Cell Signaling	#39749	AB_2799160	1:1000
P-PI3K	Cell Signaling	#17366	AB_2895293	1:1000
PI3K	Cell Signaling	#4257	AB_659889	1:1000
P-Akt	Cell Signaling	#4060	AB_2315049	1:1000
Akt	Cell Signaling	#4691	AB_915783	1:1000
P-mTOR	Cell Signaling	#5536	AB_10691552	1:1000
mTOR	Cell Signaling	#2983	AB_2105622	1:1000
2nd anti-mouse	Cell Signaling	#7076	AB_330924	1:5000
2nd anti-rabbit	Cell Signaling	#7074	AB_2099233	1:5000

## Data Availability

The data are contained within the article.
